# Vaccine effectiveness of ChAdOx1 nCoV-19 against COVID-19 in a socially vulnerable community in Rio de Janeiro, Brazil: a test-negative design study

**DOI:** 10.1016/j.cmi.2022.01.032

**Published:** 2022-05

**Authors:** Otavio T. Ranzani, Amanda A.B. Silva, Igor T. Peres, Bianca B.P. Antunes, Thiago W. Gonzaga-da-Silva, Daniel R. Soranz, José Cerbino-Neto, Silvio Hamacher, Fernando A. Bozza

**Affiliations:** 1) Barcelona Institute for Global Health, ISGlobal, Universitat Pompeu Fabra, CIBER Epidemiología y Salud Pública, Barcelona, Spain; 2) Pulmonary Division, Heart Institute (InCor), Hospital das Clinicas HCFMUSP, Faculdade de Medicina, Universidade de Sao Paulo, Sao Paulo, Brazil; 3) Department of Industrial Engineering (DEI) and Tecgraf Institute, Pontifical Catholic University of Rio de Janeiro (PUC-Rio), Rio de Janeiro, Brazil; 4) Rio de Janeiro Municipal Health Department, Rio de Janeiro, Brazil; 5) Public Health National School, Oswaldo Cruz Foundation (INI-FIOCRUZ), Rio de Janeiro, Brazil; 6) National Institute of Infectious Disease Evandro Chagas, Oswaldo Cruz Foundation (INI-FIOCRUZ), Rio de Janeiro, Brazil; 7) D’Or Institute for Research and Education (IDOR), Rio de Janeiro, Brazil

**Keywords:** Brazil, COVID-19, Test-negative, Vaccine, Vulnerable community

## Abstract

**Objectives:**

To estimate vaccine effectiveness after the first and second dose of ChAdOx1 nCoV-19 against symptomatic COVID-19 and infection in a socially vulnerable community in Brazil when Gamma and Delta were the predominant variants circulating.

**Methods:**

We conducted a test-negative study in the community Complexo da Maré, the largest group of slums (*n* = 16) in Rio de Janeiro, Brazil, from January 17, 2021 to November 27, 2021. We selected RT-qPCR positive and negative tests from a broad community testing program. The primary outcome was symptomatic COVID-19 (positive RT-qPCR test with at least one symptom) and the secondary outcome was infection (any positive RT-qPCR test). Vaccine effectiveness was estimated as 1 – OR, which was obtained from adjusted logistic regression models.

**Results:**

We included 10 077 RT-qPCR tests (6,394, 64% from symptomatic and 3,683, 36% from asymptomatic individuals). The mean age was 40 (SD: 14) years, and the median time between vaccination and RT-qPCR testing among vaccinated was 41 (25–75 percentile: 21–62) days for the first dose and 36 (25–75 percentile: 17–59) days for the second dose. Adjusted vaccine effectiveness against symptomatic COVID-19 was 31.6% (95% CI, 12.0–46.8) 21 days after the first dose and 65.1% (95% CI, 40.9–79.4) 14 days after the second dose. Adjusted vaccine effectiveness against COVID-19 infection was 31.0% (95% CI, 12.7–45.5) 21 days after the first dose and 59.0% (95% CI, 33.1–74.8) 14 days after the second dose.

**Discussion:**

ChAdOx1 nCoV-19 was effective in reducing symptomatic COVID-19 in a socially vulnerable community in Brazil when Gamma and Delta were the predominant variants circulating.

## Introduction

The impact of coronavirus disease 2019 (COVID-19) is disproportionate on socially vulnerable communities [[1], [2], [3]], which have decreased resilience when confronted by external stresses [4]. Large populations in low- and middle-income countries live in slums or favelas, which are densely populated urban areas with deteriorated or incomplete infrastructure, high risk of infectious diseases transmission, and limited access to health care services and vaccination [5].

Studies that estimate vaccine effectiveness (VE) in neighbourhoods such as favelas are lacking. Brazil has faced one of the worst public health crises worldwide because of COVID-19, which was aggravated by the spread of variants of concern (VoCs), particularly Gamma in 2021 [6]. We estimated the VE of ChAdOx1 nCoV-19 (AstraZeneca/Oxford vaccine, hereafter ChAdOx1) against symptomatic COVID-19 and infection using a test-negative design in an adult population from a large, socially vulnerable community (Complexo da Maré) in Rio de Janeiro, Brazil.

## Methods

Complexo da Maré is the largest group of favelas in Rio de Janeiro, composed of 16 favelas with 140 000 residents [7], with 54% of the population age ≤30 years and a low human development index (0.686; 123rd of Rio's 126 neighbourhoods) in 2010 [8]. From the beginning of the pandemic until November 27, 2021, the region presented high rates of positive cases (7852 of 100 000) and deaths (271 of 100 000) [9]. In July 2020, a community broad testing strategy became available at the Complexo da Maré after an effort of civil society, nongovernmental organizations, and the local community [10]. Testing was free of charge and available in tents located in three different regions in Maré. There have been 213 RT-qPCR tests per 1000 inhabitants since the beginning of the campaign. During the first period, Gamma was the prevalent VoC in Rio de Janeiro, and Delta became dominant after July 2021 (Fig. S1) [11]. This study was approved by the national research ethics committee (CAAE: 49726921.6.0000.5248).

The COVID-19 vaccination campaign in Complexo da Maré initially followed the Rio strategy starting on January 17, 2021 and according to an age-based priority, and by the end of July 2021, 38% of Maré residents had received a first dose. Maré received a mass vaccination campaign, which administered approximately 36 000 first doses of ChAdOx1 between July 29 and August 1, 2021, followed by second doses between October 14 and 16, 2021, achieving 93.4% coverage with two doses in adults (Fig. S2). Our analysis encompasses the period between January 17, 2021 and November 27, 2021. During this period, a total of 83 762 doses (64 352 first, 19 410 second, and 11 217 third doses) of ChAdOx1 were administered. We did not analyze other vaccine platforms because of the small coverage in the area. RT-qPCR tests sampled after the third dose were excluded.

We used a test-negative design to estimate the VE of ChAdOx1 against symptomatic COVID-19 (primary outcome). We linked the community-program testing database with the vaccination campaign database. Overall, we followed the methodology reported elsewhere [[12], [13], [14]]. Briefly, we selected all RT-qPCR tests (positive and negative) from symptomatic individuals, defined as presenting with at least one symptom, from RT-qPCR tests sampled within 10 days of symptom onset [13]. We excluded individuals with a previous positive RT-qPCR test and those with a negative and subsequent positive test in the following 14 days.

We estimated VE as 1 – OR from adjusted logistic regression models. Our primary analysis was effectiveness against symptomatic COVID-19 21 days after the first dose and 14 days after the second dose of ChAdOx1. We adjusted by time of epidemic (restricted cubic spline on day of the year) and subsequently adjusted by age (restricted cubic spline), sex, self-reported colour/race, Maré residence region, occupation, whether the RT-qPCR test was from routine testing, and six chronic comorbidities (cardiovascular, pulmonary, and liver diseases; diabetes; obesity; and immunosuppressed status). We evaluated the interaction between effectiveness and age groups, divided by the median of the symptomatic population (≤35 years; >35 years).

We conducted five sensitivity analyses (Table S1): (a) excluding test-negative cases that reported taste/smell alterations among symptomatic individuals [12]; (b) analyzing individuals with ≥2 symptoms; (c) analyzing symptomatic and asymptomatic cases together, (d) analyzing only asymptomatic cases, and (e) expanding the time groups after the first dose to 14 to 27, 28 to 41, 42 to 56 and >56 days. We considered the period between 0 and 13 days after the first dose as a bias indicator, because we would not expect any protection from the vaccine during this period [12]. We have missing data only for self-reported colour/race (15%), chronic comorbidities (<1%), and region of residence (<1%). We generated 20 multiple imputed datasets using chained equations. We summarized estimates using Rubin's rules. All analyses were conducted in R statistical software, version 4.0.3.

## Results

We analyzed 10,077 RT-qPCR test results after applying the inclusion and exclusion criteria (Fig. S3). Overall, 36% of tests were from asymptomatic individuals. The test positivity was 19.4% (1238 of 6394) for symptomatic and 5.7% (198 of 3485) for asymptomatic cases (Figs. S4 and S5).

The characteristics of symptomatic cases are shown in Table S2. The mean age was 38 years (standard deviation: 13 years), 65% were female, and 40% were of Brown/Pardo self-reported colour/race. The prevalence of chronic comorbidities was low. The median time between vaccination and RT-qPCR testing among vaccinated patients was 41 days (25–75 percentile: 22–62 days) for the first dose and 35 days (25–75 percentile: 18–57 days) for the second dose. The characteristics of those with ≥2 symptoms, asymptomatic and symptomatic cases combined, and asymptomatic-only cases are shown in Tables S3, S4, and S5.

VE of ChAdOx1 is shown in Table 1. Adjusted VE against symptomatic COVID-19 was 31.6% (95% CI, 12.0%–46.8%) 21 days after the first dose and 65.1% (95% CI, 40.9%–79.4%) 14 days after the second dose. The period between 0 and 13 days after the first dose (bias indicator) showed no indication of bias. After excluding negative tests from individuals with taste/smell symptoms (*n* = 5377), the adjusted VE against symptomatic COVID-19 was 65.7% (95% CI, 41.6%–79.9%) 14 days after the second dose (Table S6). When analyzing those with ≥2 symptoms (*n* = 5210), the adjusted VE against symptomatic COVID-19 was 62.3% (95% CI, 33.2%–78.8%) 14 days after the second dose (Table S7).

**Table 1:** VE of first and second doses of ChAdOx1

	Symptomatic (*n* = 6394 tests)		Symptomatic + asymptomatic (*n* = 10 077 tests)	
	OR (95% CI)	VE (95% CI)	OR (95% CI)	VE (95% CI)
**Adjusted by time of pandemic**^a^				
Unvaccinated	Reference	Reference	Reference	Reference
0–13 d after first dose	0.94 (0.62–1.43)	6% (–43.2 to 38.3)	0.82 (0.57–1.18)	18.3% (–18 to 43.4)
14–21 d after first dose	1.05 (0.67–1.64)	–4.8% (–64.1 to 33.1)	0.92 (0.62–1.37)	7.8% (–36.7 to 37.8)
>21 d after first dose	0.68 (0.54–0.87)	31.7% (13.1–46.4)	0.6 (0.48–0.75)	39.9% (25.1–51.8)
0–13 d after second dose	0.63 (0.34–1.18)	36.9% (–18.4 to 66.3)	0.62 (0.36–1.06)	38.2% (–6.1 to 63.9)
≥14 d after second dose	0.35 (0.21–0.59)	64.5% (41.2–78.6)	0.33 (0.21–0.52)	67.2% (48.1–79.2)
**Fully adjusted**^b^				
Unvaccinated	Reference	Reference	Reference	Reference
0–13 d after first dose	0.97 (0.63–1.48)	3.4% (–48 to 36.9)	0.99 (0.68–1.45)	0.9% (–45 to 32.4)
14–21 d after first dose	1.03 (0.65–1.62)	–2.9% (–62.2 to 34.8)	1.07 (0.71–1.62)	–7.3% (–61.6 to 28.8)
>21 d after first dose	0.68 (0.53–0.88)	31.6% (12.0–46.8)	0.69 (0.55–0.87)	31.0% (12.7–45.5)
0–13 d after second dose	0.67 (0.35–1.27)	33.1% (–27.1 to 64.8)	0.82 (0.47–1.44)	17.9% (–43.9 to 53.1)
≥14 d after second dose	0.35 (0.21–0.59)	65.1% (40.9–79.4)	0.41 (0.25–0.67)	59% (33.1–74.8)
**Effect modification by age (fully adjusted)**^c^				
>21 d after first dose: <35 y	0.62 (0.42–0.9)	38.5% (9.8–58.1)	0.54 (0.38–0.78)	45.7% (22.3–62)
>21 d after first dose: ≥35y	0.73 (0.55–0.98)	26.8% (2.1–45.3)	0.73 (0.56–0.95)	26.6% (4.6–43.6)
≥14 d after second dose: <35 y	0.11 (0.03–0.37)	89.2% (63–96.8)	0.10 (0.03–0.34)	89.8% (65.7–97.0)
≥14 d after second dose: ≥35 y	0.44 (0.26–0.77)	55.6% (23.2–74.3)	0.46 (0.28–0.75)	54.1% (25.1–71.9)
**Daysafter first dose before second dose (fully adjusted)**				
Unvaccinated				
0–13 d after first dose	0.99 (0.65–1.52)	0.6% (–52.3 to 35.1)	1.02 (0.69–1.49)	–1.6% (–48.9 to 30.6)
14–27 d after first dose	0.94 (0.66–1.34)	6.2% (–34 to 34.4)	0.95 (0.69–1.32)	5% (–31.6 to 31.4)
28–41 d after first dose	0.64 (0.42–0.97)	36.4% (3.2–58.2)	0.62 (0.41–0.92)	38.4% (7.8–58.9)
42–55 d after first dose	0.47 (0.29–0.76)	53.2% (24.3–71)	0.5 (0.32–0.78)	49.9% (21.7–67.9)
>56 d after first dose	0.76 (0.53–1.07)	24.5% (–7 to 46.7)	0.76 (0.55–1.05)	23.8% (–5.5 to 44.9)

VE, vaccine effectiveness.

a Adjusted by day of the year of RT-PCR-qPCR testing (restricted cubic spline).

b Adjusted by age (restricted cubic spline), sex, cardiovascular disease, respiratory disease, obesity, diabetes mellitus, immunosuppressed status (including cancer), liver disease, occupation, region of residence, self-reported race, reason of testing, and day of the year of RT-qPCR testing using a restricted cubic spline. The fully adjusted model for symptomatic and asymptomatic was adjusted by a dummy variable of symptomatic/asymptomatic.

c The p-value for interaction was 0.03 for symptomatic cases and 0.04 for symptomatic and asymptomatic cases. The reference group for the vaccine effectiveness estimates was those unvaccinated.

The young group showed higher effectiveness (Table 1). The adjusted VE increased for the subsequent days after the first dose, except for >56 days. VE when considering symptomatic and asymptomatic cases together was comparable with the main analysis (Table 1). Adjusted VE among asymptomatic cases was 26.6% (95% CI, –53.8 to –65.0%; Table S8) 21 days after the first dose.

## Discussion

We observed 31% protection after the first dose and 65% after the second dose of ChAdOx1 against symptomatic COVID-19 in a socially vulnerable community in Rio de Janeiro, Brazil, in a period of mixed Gamma and Delta variant dominance. Our estimates are in accordance with studies of ChAdOx1 effectiveness in the context of Gamma/Delta variants [14,15]. We observed that VE increased up to 53.2% during 42 to 55 days after the first dose [14] and decreased afterward in those who did not receive the second dose. The reason for the decrease in effectiveness is not clear. We can hypothesize that this decrease might occur in part because of an increase in Delta dominance and then waning, and it reinforces the need for second dose uptake.

There is limited evidence for protection against infection. Our estimates are comparable to protection against symptomatic cases [15]. However, the low number of events among asymptomatic cases shifts the estimate of the combined analysis toward symptomatic cases. Additionally, there might be some residual confounding related to reasons for being tested when asymptomatic. A detailed follow-up on asymptomatic cases could help our understanding of VE against infection.

Our study has limitations. We could not evaluate VE against COVID-19 severity. Although the test-negative design can deal with important confounding factors, such as health-seeking behaviour, we cannot rule out residual confounding for other factors (e.g. infection risk exposure) [[12], [13], [14]]. Finally, the estimates might not be generalizable to the entire population, because we analyzed only tested individuals [[12], [13], [14]].

ChAdOx1 was effective in reducing symptomatic COVID-19 in an overall young socially vulnerable community in a group of favelas in Brazil, predominantly during Gamma/Delta variant circulation. New VoCs are likely to spread (e.g. Omicron); therefore, ChAdOx1 effectiveness should be re-evaluated.

## Transparency declaration

This work is part of the Grand Challenges ICODA pilot initiative, delivered by Health Data Research UK and funded by the Bill & Melinda Gates Foundation and the Minderoo Foundation. This study was also supported by the National Council for Scientific and Technological Development, Coordinating Agency for Advanced Training of Graduate Personnel (finance code 001), Carlos Chagas Filho Foundation for Research Support of the State of Rio de Janeiro, and Pontifical Catholic University of Rio de Janeiro. OTR is funded by a Sara Borrell grant from the Instituto de Salud Carlos III (CD19/00110).

All authors reported no conflicts. All authors conducted the research independently of the funding bodies. The findings and conclusions of this article reflect the opinions of the authors and not those of the funding bodies or other affiliations of the authors.

## Author contributions

OTR, AABS, ITP, BBPA, JC, SH, and FAB conceptualized and participated in the design of the study. Conduct of the study and data collection were performed by OTR, AABS, ITP, BBPA, TWG, DRS, JC, SH, and FAB. Data analysis was performed by OTR, AABS, ITP, and BBPA. OTR drafted the initial manuscript, and all authors revised subsequent drafts. All authors read and approved the final manuscript for submission.
