# Supplementary material for: Vaccine effectiveness of ChAdOx1 nCoV-19 against COVID-19 in a socially vulnerable community in Rio de Janeiro, Brazil: a test-negative design study
Source: Clin Microbiol Infect. 2022 May;28(5):736.e1–4. doi: 10.1016/j.cmi.2022.01.032 (PMC8828302; doi:10.1016/j.cmi.2022.01.032)
Supplement: Multimedia component 1 [file mmc1.pdf]

Supplementary Data

Vaccine Effectiveness of ChAdOx1 nCoV-19 Against COVID-19 in a Socially Vulnerable Community in Rio de Janeiro, Brazil: a test-negative design study

Table of Contents

eFigure 1. Variants of Interest and Concern in Rio de Janeiro State from Jan to Nov 2021 . 2

eFigure 2. First and second doses of AstraZeneca vaccine during the vaccination campaign in the “Complexo da Maré” 3

eTable 1. Description of the population for the main and sensitivity analyses 4

eFigure 3. Flowchart 5

eFigure 4. RT-qPCR tests and results during the study period stratified by presence of symptoms 6

eFigure 5. Test positivity proportion during the study period stratified by presence of symptoms 7

eTable 2 - Characteristics for the cases with at least 1 symptom 8

eTable 3 - Characteristics for the cases with at least 2 symptoms 9

eTable 4 - Characteristics for symptomatic and asymptomatic cases together 10

eTable 5. Characteristics for the asymptomatic cases 11

eTable 6. Vaccine effectiveness against symptomatic COVID-19 (sensitivity analysis 1 excluding test-negatives with taste/smell symptoms) 12

eTable 7. Vaccine effectiveness against symptomatic COVID-19 (sensitivity analysis 2, at least 2 symptoms) 13

eTable 8. Vaccine effectiveness against asymptomatic COVID-19 (sensitivity analysis 4) . 14

eFigure 1. Variants of Interest and Concern in Rio de Janeiro State from Jan to Nov 2021

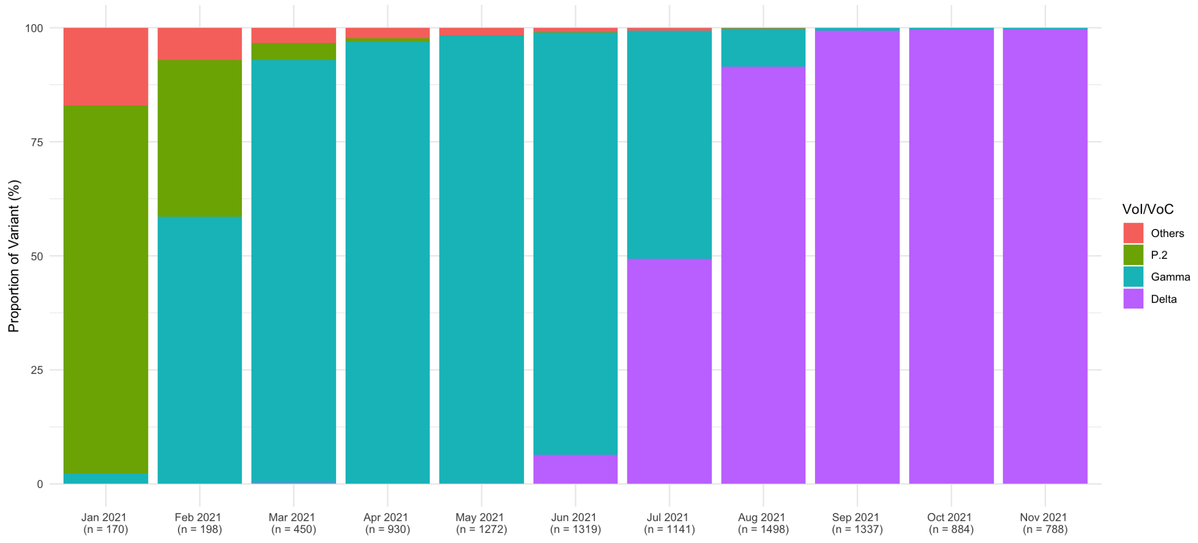

eFigure 2. First and second doses of AstraZeneca vaccine during the vaccination campaign in the “Complexo da Maré”

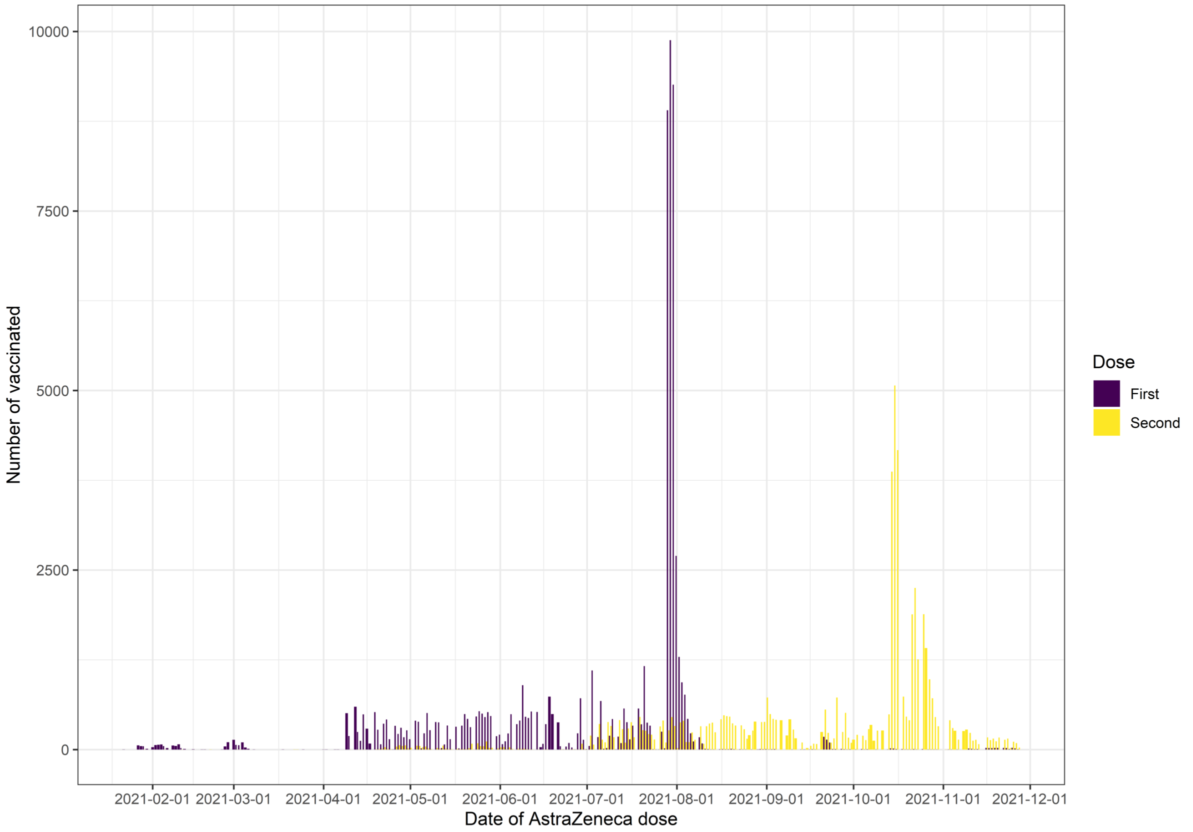

eTable 1. Description of the population for the main and sensitivity analyses

Analysis	Population	Label	Vaccine status
Main (Table 1)	At least 1 symptom	Symptomatic	Unvaccinated, 0-13, 14-21, ≥21 days after first dose, 0-13, ≥14 days after second dose
Sensitivity 1 (eTable)	At least 1 symptom, excluding test-negative cases that reported taste/smell alterations	Symptomatic after excluding negative tests from individuals with taste/smell symptoms	Unvaccinated, 0-13, 14-21, ≥21 days after first dose, 0-13, ≥14 days after second dose
Sensitivity 2 (eTable 2)	At least 2 symptoms	Symptomatic-2	Unvaccinated, 0-13, 14-21, ≥21 days after first dose, 0-13, ≥14 days after second dose
Sensitivity 3 (Table 1)	Tested independent of symptoms (at least 1 symptom and asymptomatic)	Symptomatic and Asymptomatic	Unvaccinated, 0-13, 14-21, ≥21 days after first dose, 0-13, ≥14 days after second dose
Sensitivity 4 (eTable 8)	Asymptomatic	Asymptomatic	Unvaccinated, 0-13, 14-21, ≥21 days after first dose, 0-13, ≥14 days after second dose
Sensitivity 5 (Table 1)	At least 1 symptom / tested independent of symptoms (at least 1 symptom and asymptomatic)	-	Unvaccinated, 0-13, 14-27, 28-41, 42-56 and >56 days after first dose

eFigure 3. Flowchart

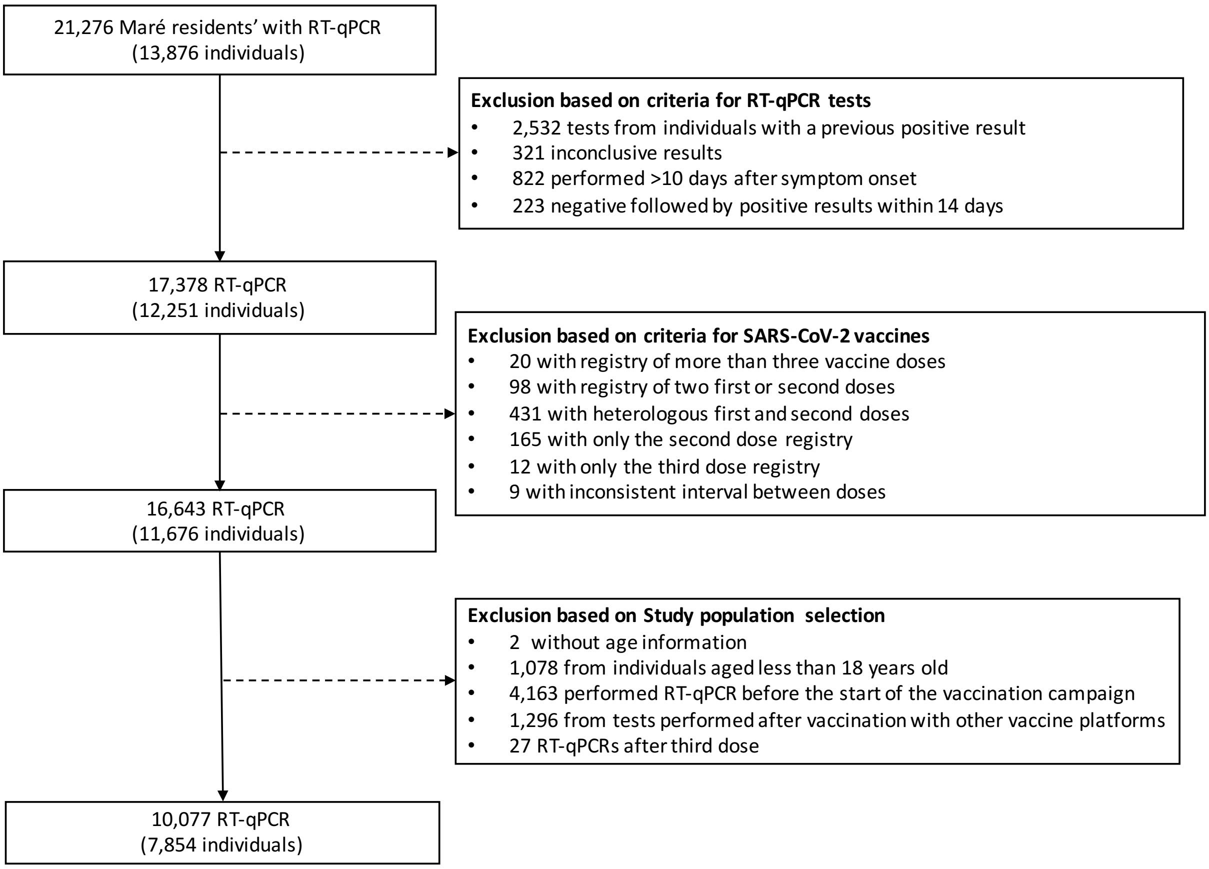

eFigure 4. RT-qPCR tests and results during the study period stratified by presence of symptoms

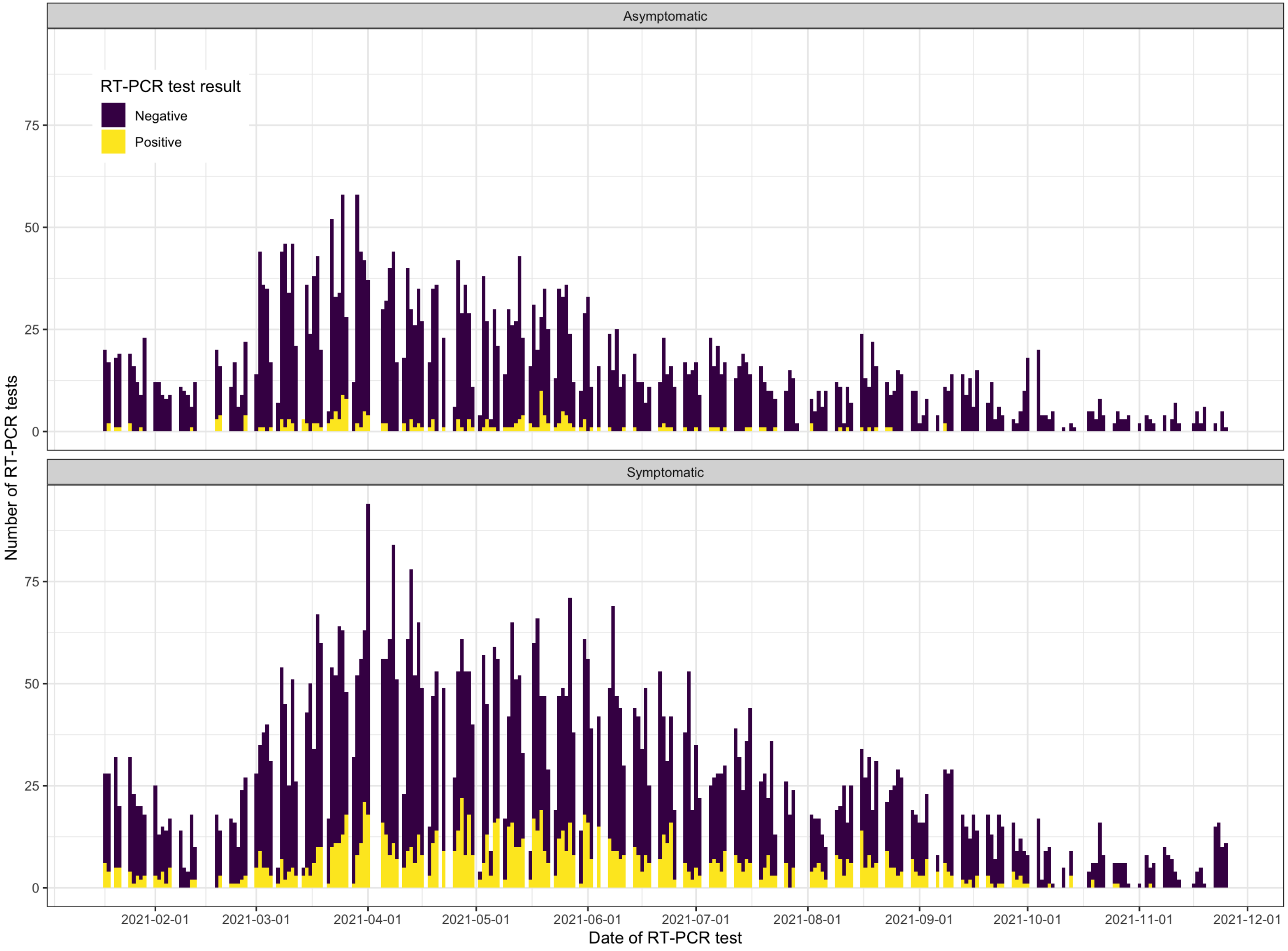

eFigure 5. Test positivity proportion during the study period stratified by presence of symptoms

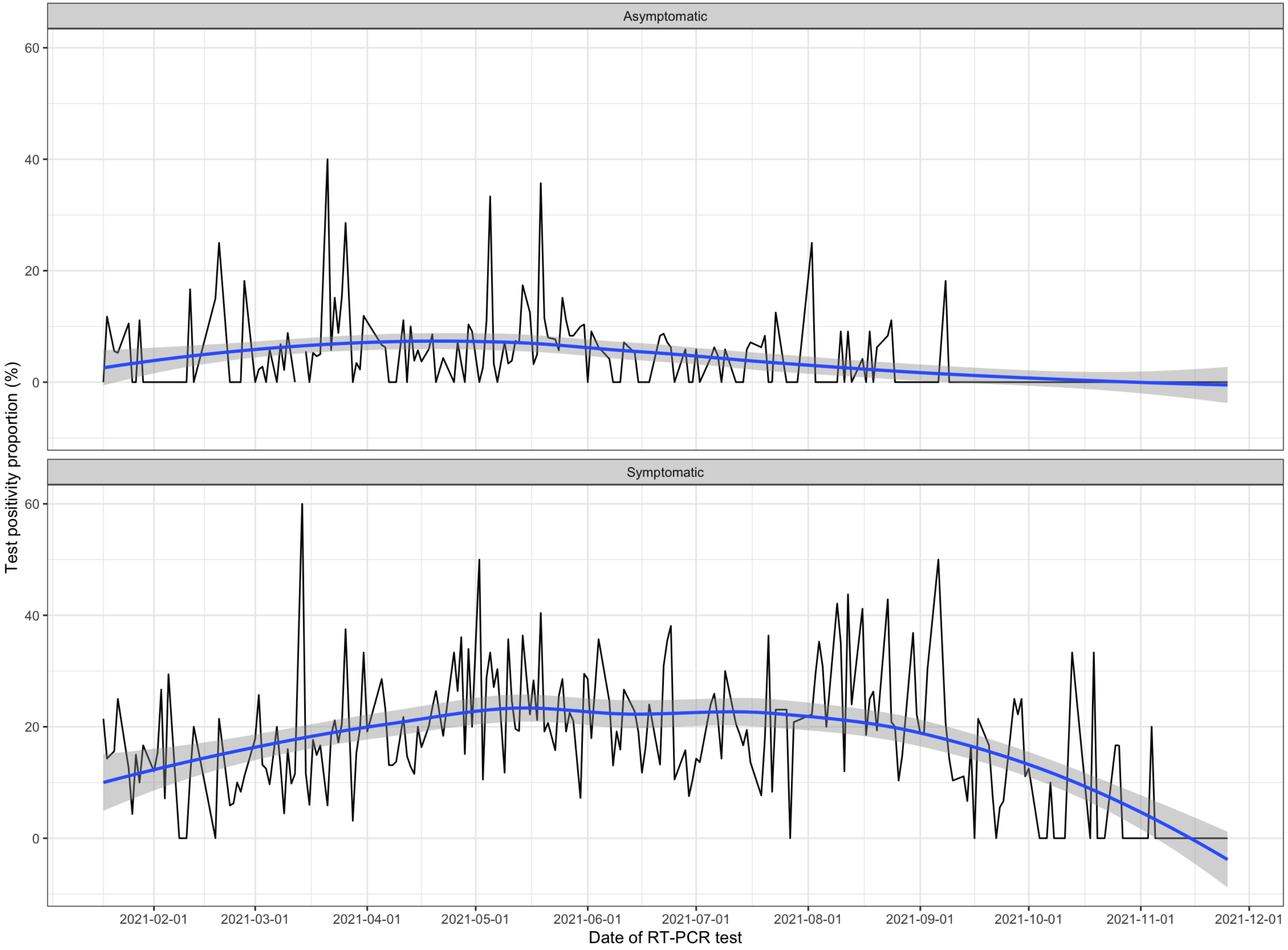

The blue line represents the smooth curve and shade area the 95% confidence intervals

eTable 2 - Characteristics for the cases with at least 1 symptom

	Overall (n=6,394)	Test Negative (n=5,156)	Test Positive (n=1,238)
Age, mean ± SD	38.2 (13)	38.2 (13)	38.5 (14)
Age ≤35 years	3051 (47.7)	2457 (47.7)	594 (48.0)
Age >35 years	3343 (52.3)	2699 (52.3)	644 (52.0)
Sex			
Female	4165 (65.1)	3417 (66.3)	748 (60.4)
Male	2229 (34.9)	1739 (33.7)	490 (39.6)
Self-reported race			
White	1571 (24.6)	1274 (24.7)	297 (24.0)
Brown	2553 (39.9)	2071 (40.2)	482 (38.9)
Black	1116 (17.5)	913 (17.7)	203 (16.4)
Other	166 (2.6)	138 (2.7)	28 (2.3)
Missing	955 (14.9)	731 (14.2)	224 (18.1)
Comorbidities*			
Cardiovascular disease	482 (7.5)	405 (7.9)	77 (6.2)
Respiratory disease	192 (3.0)	164 (3.2)	28 (2.3)
Imunossupressed status	272 (4.3)	230 (4.5)	42 (3.4)
Liver disease	104 (1.6)	82 (1.6)	22 (1.8)
Obesity	583 (9.1)	495 (9.6)	88 (7.1)
Diabetes mellitus	460 (7.2)	386 (7.5)	74 (6.0)
**Occupation**			
Other	6057 (94.7)	4863 (94.3)	1194 (96.4)
Security	78 (1.2)	67 (1.3)	11 (0.9)
Healthcare worker	259 (4.1)	226 (4.4)	33 (2.7)
**Region of residence**			
Centre	3555 (55.6)	2918 (56.6)	637 (51.5)
North	417 (6.5)	329 (6.4)	88 (7.1)
South	2405 (37.6)	1893 (36.7)	512 (41.4)
Missing	17 (0.3)	16 (0.3)	1 (0.1)
**Vaccination status**			
**Main analysis**			
Unvaccinated	4928 (77.1)	3921 (76.0)	1007 (81.3)
0-13 days after first dose	133 (2.1)	103 (2.0)	30 (2.4)
14-21 days after first dose	110 (1.7)	83 (1.6)	27 (2.2)
>21 days after first dose	799 (12.5)	665 (12.9)	134 (10.8)
0-13 days after second dose	88 (1.4)	75 (1.5)	13 (1.1)
≥14 days after second dose	336 (5.3)	309 (6.0)	27 (2.2)
**Sensitivity analysis^**			
Unvaccinated	4928 (82.5)	3921 (82.2)	1007 (84.1)
0-13 days after first dose	133 (2.2)	103 (2.2)	30 (2.5)
14-27 days after first dose	203 (3.4)	158 (3.3)	45 (3.8)
28-41 days after first dose	186 (3.1)	156 (3.3)	30 (2.5)
42-55 days after first dose	178 (3.0)	156 (3.3)	22 (1.8)
>56 days after first dose	342 (5.7)	278 (5.8)	64 (5.3)
**Time between first dose and PCR, median [p25, p75]**	41 [22, 62]	42 [23, 62]	36 [19, 62]
**Time between second dose and PCR, median [p25, p75]**	35 [18, 57]	35 [18, 57]	34 [9, 61]

* 4 missing values for each comorbidity. ^Includes 5,970 RT-qPCRs for the first dose.
Data as N (%) unless differently reported

eTable 3 - Characteristics for the cases with at least 2 symptoms

	Overall (n=5,210)	Test Negative (n=4,113)	Test Positive (n=1,097)
Age, mean ± SD	37.7 (13)	37.6 (13)	38.2 (13)
Age ≤35 years	2576 (49.4)	2043 (49.7)	533 (48.6)
Age >35 years	2634 (50.6)	2070 (50.3)	564 (51.4)
Sex			
Female	3453 (66.3)	2785 (67.7)	668 (60.9)
Male	1757 (33.7)	1328 (32.3)	429 (39.1)
Self-reported race			
White	1326 (25.5)	1053 (25.6)	273 (24.9)
Brown	2130 (40.9)	1701 (41.4)	429 (39.1)
Black	922 (17.7)	738 (17.9)	184 (16.8)
Other	142 (2.7)	115 (2.8)	27 (2.5)
Missing	670 (12.9)	488 (11.9)	182 (16.6)
Comorbidities*			
Cardiovascular disease	382 (7.3)	314 (7.6)	68 (6.2)
Respiratory disease	170 (3.3)	143 (3.5)	27 (2.5)
Imunossupressed status	234 (4.5)	199 (4.8)	35 (3.2)
Liver disease	91 (1.7)	71 (1.7)	20 (1.8)
Obesity	497 (9.5)	415 (10.1)	82 (7.5)
Diabetes mellitus	361 (6.9)	297 (7.2)	64 (5.8)
**Occupation**			
Other	4933 (94.7)	3876 (94.2)	1057 (96.4)
Security	60 (1.2)	51 (1.2)	9 (0.8)
Healthcare worker	217 (4.2)	186 (4.5)	31 (2.8)
**Region of residence**			
Centre	2908 (55.8)	2339 (56.9)	569 (51.9)
North	323 (6.2)	248 (6.0)	75 (6.8)
South	1964 (37.7)	1512 (36.8)	452 (41.2)
Missing	15 (0.3)	14 (0.3)	1 (0.1)
**Vaccination status**			
**Main analysis**			
Unvaccinated	4040 (77.5)	3144 (76.4)	896 (81.7)
0-13 days after first dose	102 (2.0)	76 (1.8)	26 (2.4)
14-21 days after first dose	88 (1.7)	65 (1.6)	23 (2.1)
>21 days after first dose	646 (12.4)	528 (12.8)	118 (10.8)
0-13 days after second dose	69 (1.3)	59 (1.4)	10 (0.9)
≥14 days after second dose	265 (5.1)	241 (5.9)	24 (2.2)
**Sensitivity analysis^**			
Unvaccinated	4040 (82.9)	3144 (82.5)	896 (84.3)
0-13 days after first dose	102 (2.1)	76 (2.0)	26 (2.4)
14-27 days after first dose	167 (3.4)	128 (3.4)	39 (3.7)
28-41 days after first dose	154 (3.2)	129 (3.4)	25 (2.4)
42-55 days after first dose	142 (2.9)	123 (3.2)	19 (1.8)
>56 days after first dose	271 (5.6)	213 (5.6)	58 (5.5)
**Time between first dose and PCR, median [p25, p75]**	41 [22, 62]	42 [23, 62]	38 [19, 63]
**Time between second dose and PCR, median [p25, p75]**	35 [18-58]	34 [18-56]	36 [12-62]

* 2 missing values for each comorbidity. ^Includes 4,876 RT-qPCRs for the first dose.
Data as N (%) unless differently reported

eTable 4 - Characteristics for symptomatic and asymptomatic cases together

	Overall (n=10,077)	Test Negative (n=8,641)	Test Positive (n=1,436)
Age, mean ± SD	40 (14)	40.1 (14)	39.1 (14)
Age ≤35 years	4334 (43.0)	3670 (42.5)	664 (46.2)
Age >35 years	5743 (57.0)	4971 (57.5)	772 (53.8)
Sex			
Female	6380 (63.3)	5510 (63.8)	870 (60.6)
Male	3697 (36.7)	3131 (36.2)	566 (39.4)
Self-reported race			
White	2236 (22.2)	1904 (22.0)	332 (23.1)
Brown	3796 (37.7)	3245 (37.6)	551 (38.4)
Black	1891 (18.8)	1647 (19.1)	244 (17.0)
Other	271 (2.7)	238 (2.8)	33 (2.3)
Missing	1783 (17.7)	1511 (17.5)	272 (18.9)
Comorbidities*			
Cardiovascular disease	781 (7.8)	691 (8.0)	90 (6.3)
Respiratory disease	240 (2.4)	209 (2.4)	31 (2.2)
Imunossupressed status	364 (3.6)	319 (3.7)	45 (3.1)
Liver disease	132 (1.3)	110 (1.3)	22 (1.5)
Obesity	757 (7.5)	664 (7.7)	93 (6.5)
Diabetes mellitus	690 (6.8)	610 (7.1)	80 (5.6)
**Occupation**			
Other	9588 (95.1)	8200 (94.9)	1388 (96.7)
Security	119 (1.2)	107 (1.2)	12 (0.8)
Healthcare worker	370 (3.7)	334 (3.9)	36 (2.5)
**Region of residence**			
Centre	5584 (55.4)	4844 (56.1)	740 (51.5)
North	745 (7.4)	643 (7.4)	102 (7.1)
South	3725 (37.0)	3132 (36.2)	593 (41.3)
Missing	23 (0.2)	22 (0.3)	1 (0.1)
**Vaccination status**			
**Main analysis**			
Unvaccinated	7684 (76.3)	6509 (75.3)	1175 (81.8)
0-13 days after first dose	232 (2.3)	196 (2.3)	36 (2.5)
14-21 days after first dose	185 (1.8)	153 (1.8)	32 (2.2)
>21 days after first dose	1254 (12.4)	1109 (12.8)	145 (10.1)
0-13 days after second dose	153 (1.5)	136 (1.6)	17 (1.2)
≥14 days after second dose	569 (5.6)	538 (6.2)	31 (2.2)
**Sensitivity analysis^**			
Unvaccinated	7684 (82.1)	6509 (81.7)	1175 (84.7)
0-13 days after first dose	232 (2.5)	196 (2.5)	36 (2.6)
14-27 days after first dose	341 (3.6)	289 (3.6)	52 (3.7)
28-41 days after first dose	273 (2.9)	242 (3.0)	31 (2.2)
42-55 days after first dose	275 (2.9)	250 (3.1)	25 (1.8)
>56 days after first dose	550 (5.9)	481 (6.0)	69 (5.0)
**Time between first dose and PCR, median [p25, p75]**	41 [21, 62]	42 [21, 62]	36 [18, 62]
**Time between second dose and PCR, median [p25, p75]**	36 [17-59]	37 [18-59]	29 [9-59]

* 7 missing values for each comorbidity. ^Includes 9,355 RT-qPCRs for the first dose.
Data as N (%) unless differently reported.

eTable 5. Characteristics for the asymptomatic cases

	Overall (n=3,683)	Test Negative (n=3,485)	Test Positive (n=198)
Age, mean ± SD	42.9 (15)	42.9 (15)	43.0 (15)
Age ≤35 years	1283 (34.8)	1213 (34.8)	70 (35.4)
Age >35 years	2400 (65.2)	2272 (65.2)	128 (64.6)
Sex			
Female	2215 (60.1)	2093 (60.1)	122 (61.6)
Male	1468 (39.9)	1392 (39.9)	76 (38.4)
Self-reported race			
White	665 (18.1)	630 (18.1)	35 (17.7)
Brown/Pardo	1243 (33.7)	1174 (33.7)	69 (34.8)
Black	775 (21.0)	734 (21.1)	41 (20.7)
Other	105 (2.9)	100 (2.9)	5 (2.5)
Missing	828 (22.5)	780 (22.4)	48 (24.2)
Comorbidities*			
Cardiovascular disease	299 (8.1)	286 (8.2)	13 (6.6)
Respiratory disease	48 (1.3)	45 (1.3)	3 (1.5)
Imunossupressed status	92 (2.5)	89 (2.6)	3 (1.5)
Liver disease	28 (0.8)	28 (0.8)	0 (0.0)
Obesity	174 (4.7)	169 (4.8)	5 (2.5)
Diabetes mellitus	230 (6.2)	224 (6.4)	6 (3.0)
**Occupation**			
Other	3531 (95.9)	3337 (95.8)	194 (98.0)
Security	41 (1.1)	40 (1.1)	1 (0.5)
Healthcare worker	111 (3.0)	108 (3.1)	3 (1.5)
**Region of residence**			
Centre	2029 (55.1)	1926 (55.3)	103 (52.0)
North	328 (8.9)	314 (9.0)	14 (7.1)
South	1320 (35.8)	1239 (35.6)	81 (40.9)
Missing	6 (0.2)	6 (0.2)	0 (0.0)
**Vaccination status**			
**Main analysis**			
Unvaccinated	2756 (74.8)	2588 (74.3)	168 (84.8)
0-13 days after first dose	99 (2.7)	93 (2.7)	6 (3.0)
14-21 days after first dose	75 (2.0)	70 (2.0)	5 (2.5)
>21 days after first dose	455 (12.4)	444 (12.7)	11 (5.6)
0-13 days after second dose	65 (1.8)	61 (1.8)	4 (2.0)
≥14 days after second dose	233 (6.3)	229 (6.6)	4 (2.0)
**Sensitivity analysis^**			
Unvaccinated	2756 (81.4)	2588 (81.0)	168 (88.4)
0-13 days after first dose	99 (2.9)	93 (2.9)	6 (3.2)
14-27 days after first dose	138 (4.1)	131 (4.1)	7 (3.7)
28-41 days after first dose	87 (2.6)	86 (2.7)	1 (0.5)
42-55 days after first dose	97 (2.9)	94 (2.9)	3 (1.6)
>56 days after first dose	208 (6.1)	203 (6.4)	5 (2.6)
**Time between first dose and PCR, median [p25, p75]**	40 [19, 62]	41 [20, 63]	21 [12, 53]
**Time between second dose and PCR, median [p25, p75]**	38 [15-60]	39 [18-61]	13 [6-34]

* 3 missing value for each comorbidity. ^Includes 3,385 RT-qPCRs for the first dose.
Data as N (%) unless differently reported.

eTable 6. Vaccine effectiveness against symptomatic COVID-19 (sensitivity analysis 1 excluding test-negatives with taste/smell symptoms**)**

	**Symptomatic – S1 (n = 5,377 tests)**	
	**OR (95% CI)**	**VE (95% CI)**
**Adjusted by time of pandemic***		
Unvaccinated	Reference	Reference
0-13 days after first dose	0.90 (0.59-1.39)	9.9% (-38.6-41.5)
14-21 days after first dose	1.05 (0.66-1.66)	-4.8% (-66.3-33.9)
>21 days after first dose	0.65 (0.50-0.83)	35.4% (17.4-49.5)
0-13 days after second dose	0.60 (0.32-1.14)	39.6% (-14.5-68.1)
≥14 days after second dose	0.34 (0.21-0.57)	65.6% (42.8-79.3)
**Fully adjusted^**		
Unvaccinated	Reference	Reference
0-13 days after first dose	0.94 (0.61-1.45)	6.2% (-45.2-39.3)
14-21 days after first dose	1.05 (0.66-1.68)	-5.1% (-67.8-34.2)
>21 days after first dose	0.65 (0.50-0.84)	34.8% (15.7-49.6)
0-13 days after second dose	0.64 (0.33-1.23)	35.8% (-23.3-66.5)
≥14 days after second dose	0.34 (0.20-0.58)	65.7% (41.6-79.9)

* Adjusted by day of the year of RT-PCR testing (restricted cubic spline); ^ Adjusted by age (restricted cubic spline), sex, cardiovascular disease, respiratory disease, obesity, diabetes mellitus, immunosuppressed status (includes cancer), liver disease, occupation, region of residence, self-reported race, reason of testing, and day of the year of RT-PCR testing using a restricted cubic spline.

eTable 7. Vaccine effectiveness against symptomatic COVID-19 (sensitivity analysis 2, at least 2 symptoms**)**

	**Symptomatic – S2 (≥2 symptoms) (n = 5,210 tests)**	
	**OR (95% CI)**	**VE (95% CI)**
**Adjusted by time of pandemic***		
Unvaccinated	Reference	Reference
0-13 days after first dose	0.99 (0.63-1.58)	0.5% (-57.7-37.2)
14-21 days after first dose	1.02 (0.62-1.67)	-2.1% (-67.0-37.5)
>21 days after first dose	0.69 (0.53-0.89)	31.3% (11.0-47.0)
0-13 days after second dose	0.57 (0.28-1.17)	42.6% (-17.1-71.9)
≥14 days after second dose	0.38 (0.22-0.66)	61.7% (33.7-77.9)
**Fully adjusted^**		
Unvaccinated	Reference	Reference
0-13 days after first dose	1.03 (0.65-1.65)	-3.2% (-64.9-35.4)
14-21 days after first dose	1.02 (0.62-1.68)	-1.6% (-67.6-38.3)
>21 days after first dose	0.67 (0.51-0.87)	33.3% (12.6-49.1)
0-13 days after second dose	0.62 (0.30-1.29)	37.9% (-28.5-70.0)
≥14 days after second dose	0.38 (0.21-0.67)	62.3% (33.2-78.8)

* Adjusted by day of the year of RT-PCR testing (restricted cubic spline); ^ Adjusted by age (restricted cubic spline), sex, cardiovascular disease, respiratory disease, obesity, diabetes mellitus, immunosuppressed status (includes cancer), liver disease, occupation, region of residence, self-reported race, reason of testing, and day of the year of RT-PCR testing using a restricted cubic spline.

eTable 8. Vaccine effectiveness against asymptomatic COVID-19 (sensitivity analysis 4**)**

	**Asymptomatic – S4 (n = 3,683 tests)**	
	**OR (95% CI)**	**VE (95% CI)**
**Adjusted by time of pandemic***		
Unvaccinated	Reference	Reference
0-13 days after first dose	1.05 (0.45-2.49)	-5.4% (-148.9-55.3)
14-21 days after first dose	1.35 (0.52-3.51)	-35.3% (-250.7-47.8)
>21 days after first dose	0.64 (0.32-1.29)	36.1% (-29.3-68.4)
0-13 days after second dose	-[&]	-[&]
≥14 days after second dose	-[&]	-[&]
**Fully adjusted^**		
Unvaccinated	Reference	Reference
0-13 days after first dose	1.13 (0.47-2.72)	-12.9% (-171.6-53.1)
14-21 days after first dose	1.42 (0.54-3.75)	-42.4% (-274.9-45.9)
>21 days after first dose	0.73 (0.35-1.54)	26.6% (-53.8-65.0)
0-13 days after second dose	-[&]	-[&]
≥14 days after second dose	-[&]	-[&]

* Adjusted by day of the year of RT-PCR testing (restricted cubic spline); ^ Adjusted by age (restricted cubic spline), sex, cardiovascular disease, respiratory disease, obesity, diabetes mellitus, immunosuppressed status (includes cancer), liver disease, occupation, region of residence, self-reported race, reason of testing, and day of the year of RT-PCR testing using a restricted cubic spline. & Not enough events.
